# Serial Expression of Pro-Inflammatory Biomarkers in Acute Lung Injury During the Post-Resuscitation Periods in Rats with Cardiac Arrest

**DOI:** 10.3390/ijms27020786

**Published:** 2026-01-13

**Authors:** Han-Ping Wu, Kuan-Miao Lin, Mao-Jen Lin

**Affiliations:** 1 Department of Medicine, College of Medicine, Chang Gung University, Taoyuan 333, Taiwan; arthur1226@gmail.com; 2 Department of Pediatrics, Chiayi Chang Gung Memorial Hospital, Chiayi 613, Taiwan; cheshire@cgmh.org.tw; 3 Department of Medicine, Taichung Tzu Chi Hospital, The Buddhist Tzu Chi Medical Foundation, Taichung 427, Taiwan; 4 Department of Medicine, College of Medicine, Tzu Chi University, Hualien 970, Taiwan

**Keywords:** cardiac arrest, cardiopulmonary resuscitation, MyD88, lung injury, innate immunity

## Abstract

Acute lung injury may occur after cardiac arrest (CA), with innate immunity likely playing an important role in lung inflammation after CA. This study aimed to survey serial changes in the toll-like receptor (TLR) 4 signaling pathway in post-resuscitation lung injury in CA rats. A randomized animal study was conducted in rats with CA followed by successful cardiopulmonary resuscitation (CPR). The expression of TLR4 pathway biomarkers was analyzed and compared to the sham controls at different time points after CA with CPR. Lung tissues were collected for histological analysis to assess structural damage. Bronchoalveolar lavage fluid (BALF) was analyzed to quantify inflammatory cytokines and to assess changes in regulatory B cells (Bregs) and regulatory T cells (Tregs). Histological examination revealed marked pulmonary hemorrhage and structural injury shortly after CA. CA with CPR increased myeloid differentiation factor 88 (MyD88) mRNA and protein expression compared to controls at 2 h after CA. Cytokine analysis of BALF showed elevated IFN-γ, interleukin (IL)-1α, IL-1β, IL-2, IL-6, and IL-10 at 2 h after CA. A reduction in Bregs was noted at 2 h, whereas Tregs transiently increased between 2 and 4 h but declined at 6 h after CA. The MyD88-dependent signaling pathway appears to be rapidly activated in rats with CA after CPR, which may contribute to the early pulmonary inflammation observed as soon as 2 h after CA.

## 1. Introduction

Cardiac arrest (CA) is a critical clinical emergency with extremely high mortality. Timely and accurate cardiopulmonary resuscitation (CPR) remains the most crucial factor in determining patient survival and prognosis [1]. Although CPR plays an irreplaceable role in restoring circulation, related evidence suggests that chest compressions, while lifesaving, may inadvertently cause mechanical injury to vital organs. The lungs appear to be especially vulnerable, with lung damage accompanied by inflammatory responses observed in approximately one-third of successfully resuscitated patients [2]. These injuries may not only compromise pulmonary gas exchange but also exacerbate systemic inflammation, thereby contributing to poor post-resuscitation outcomes.

Among the many signaling pathways involved in pulmonary inflammation, the toll-like receptor (TLR) 4/myeloid differentiation primary response protein 88 (MyD88) pathway has been identified as a key mediator [3]. MyD88 acts as a central adaptor molecule in innate immune signaling and is crucial for the initiation and amplification of inflammatory responses [4]. Activation of MyD88 leads to the downstream activation of several pro-inflammatory pathways, including nuclear factor kappa B, interferon regulatory factors, and the mitogen-activated protein kinase cascade [5]. These pathways, in turn, upregulate the transcription and secretion of various cytokines and chemokines, such as interleukin (IL)-1β, IL-6, and tumor necrosis factor (TNF).

Under normal physiological conditions, the balance between pro-inflammatory and anti-inflammatory cytokines is tightly regulated by a complex network of immune cells and signaling molecules [6]. Among these, regulatory T cells (Tregs) and regulatory B cells (Bregs) play essential immunomodulatory roles. Tregs suppress the activation and proliferation of effector T cells and are vital for preventing autoimmune and excessive inflammatory responses [7]. Bregs, on the other hand, exert immunosuppressive effects mainly through the secretion of anti-inflammatory cytokines such as IL-10 and transforming growth factor-beta, thereby maintaining immune homeostasis and promoting tissue repair [8]. Although Tregs and Bregs have been well studied in inflammatory and autoimmune diseases, their dynamic roles in pulmonary inflammation after CA with CPR remain unclear. Likewise, the involvement of MyD88 signaling in post-resuscitation lung injury has not been fully elucidated. In this study, we aimed to use a CA/CPR rodent model to investigate changes in MyD88 expression, cytokine profiles, and Treg/Breg dynamics in lung tissues and bronchoalveolar lavage fluid at different time points to capture both early immune activation and later regulatory processes.

## 2. Results

**Figure 1:**
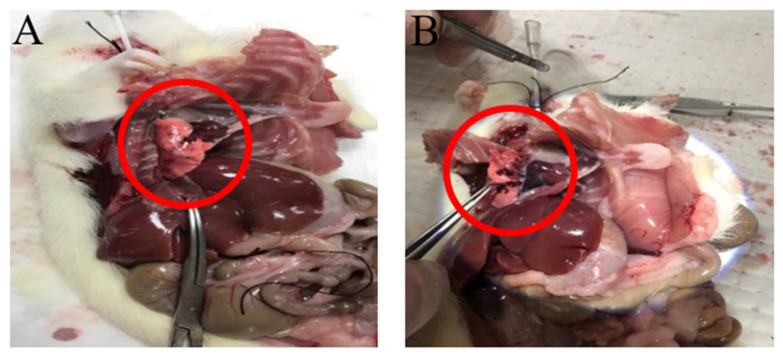
Gross findings of lung tissues. Pulmonary hemorrhage in rats (**A**) 4 and (**B**) 6 h after CA/CPR. The red circles show major areas of pulmonary hemorrhage.

**Figure 2:**
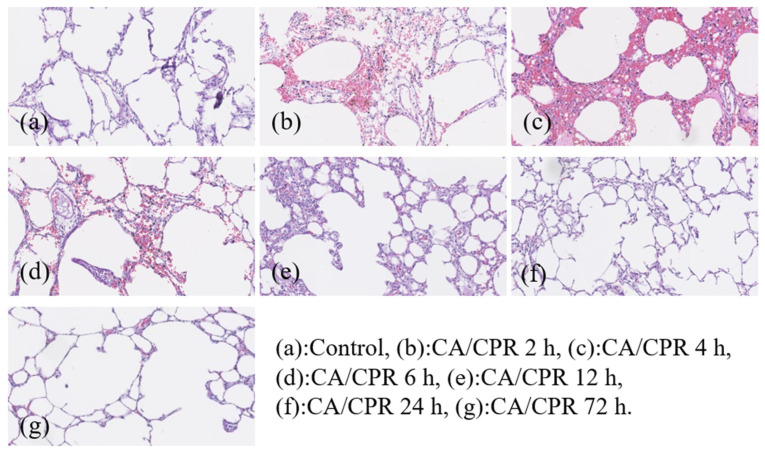
Pathological findings of lungs in rats at different time points after CA/CPR. All images were captured using the same microscope at 200× magnification.

To investigate the potential role of the MyD88-dependent signaling pathway in post-resuscitative lung injury following CA, the mRNA and protein expression levels of MyD88 were examined in successfully resuscitated rats and normal control rats. Animals were sacrificed at 2, 4, 6, 12, 24, and 72 h after CA induction, followed by successful CPR. Lung tissues were collected for further analysis to assess the temporal dynamics of MyD88 expression and its possible involvement in post-resuscitation inflammatory responses in the lungs. Gross and histological findings from the lung tissues after CA with CPR are shown in Figure 1 and Figure 2, respectively. Pulmonary hemorrhage occurred at 4 and 6 h. Compared with the control group, the resuscitated rats exhibited gradual alveolar wall thickening between 2 and 4 h, and inflammation was observed from 2 to 12 h. After 24 h of CPR, lung tissue slices returned to normal.

**Figure 3:**
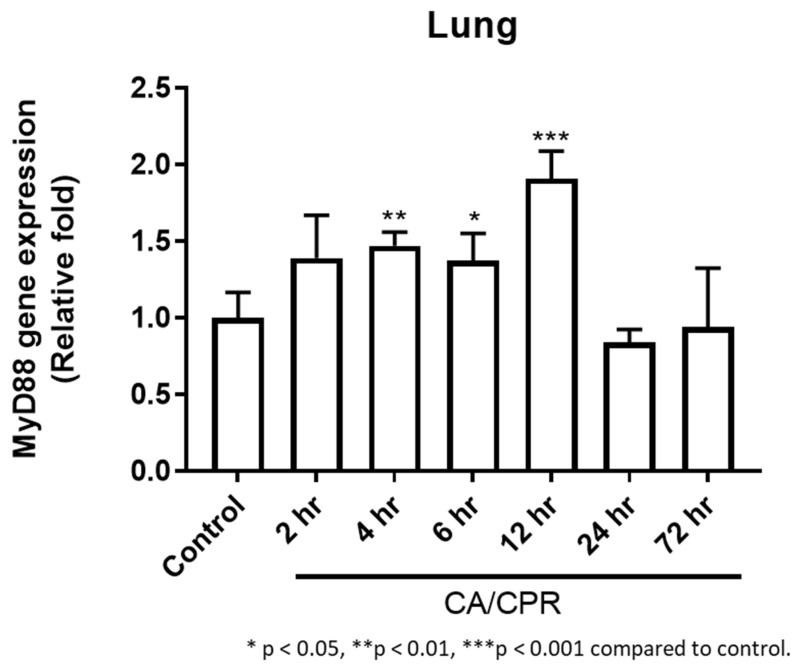
MyD88 qPCR analysis: MyD88 mRNA expression from 2 to 72 h in rats with CA/CPR.

**Figure 4:**
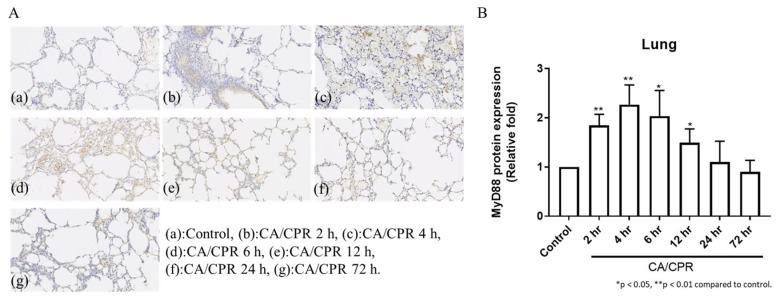
IHC staining of MyD88 protein expression of lung tissues in rats with CA/CPR. (**A**) IHC-stained images showing MyD88 expression. All images were captured using the same microscope at 200× magnification. (**B**) Quantification of MyD88 expression.

**Figure 5:**
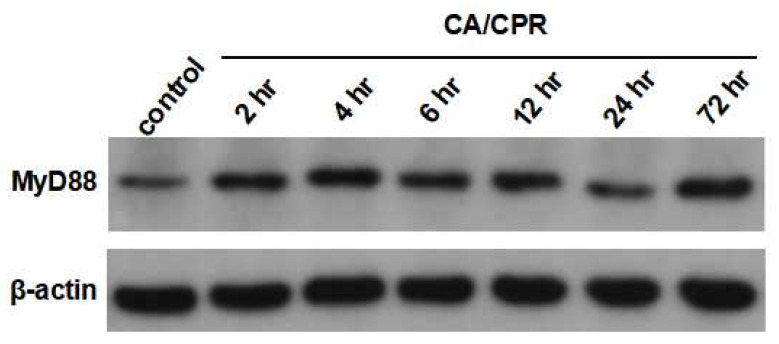
Western blotting of MyD88 protein of lung tissues in rats with CA/CPR.

MyD88 mRNA expression in lung tissues increased as early as 2 h after CA with CPR and reached a marked elevation at 12 h, followed by a decrease at 24 h (Figure 3). Correspondingly, IHC results showed that MyD88 protein levels began to rise at 2 h and were significantly elevated at 4 and 6 h, followed by a gradual decline (Figure 4). Meanwhile, Western blot analysis also showed an increase in MyD88 protein at 2 h, with a decline observed at 24 h (Figure 5), exhibiting a temporal pattern similar to that observed in IHC.

**Figure 6:**
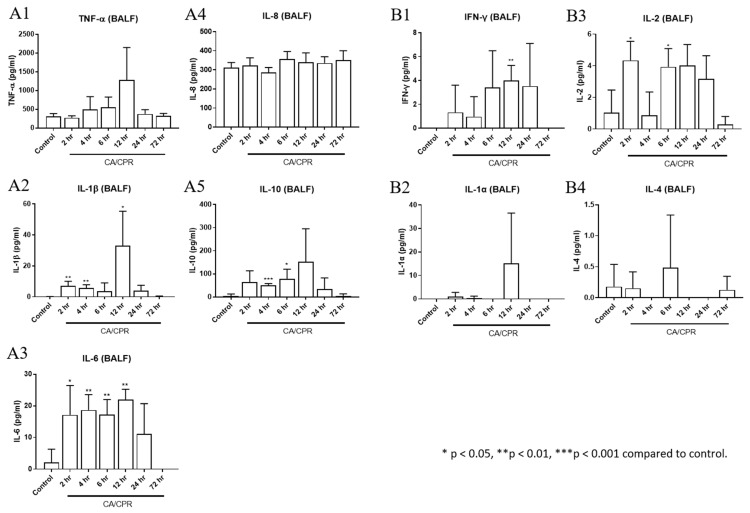
Cytokine levels of bronchoalveolar lavage fluid (BALF). The concentrations of (**A1**) TNF-α, (**A2**) IL-1β, (**A3**) IL-6, (**A4**) IL-8, and (**A5**) IL-10 were measured by enzyme-linked immunosorbent assay. The concentrations of (**B1**) IFN-γ, (**B2**) IL-1α, (**B3**) IL-2, and (**B4**) IL-4 were measured by cytometric bead array immunoassay.

**Figure 7:**
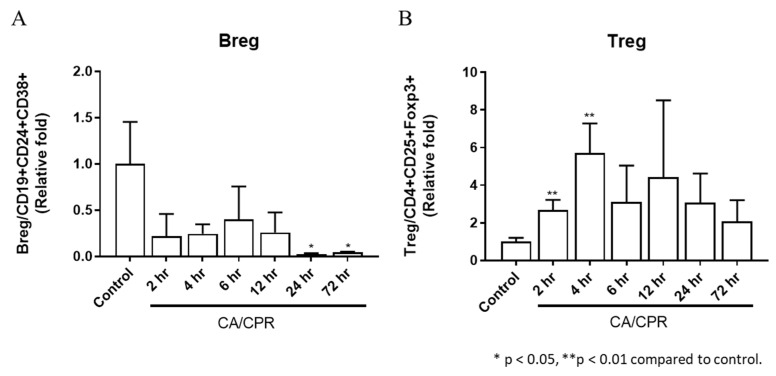
Expression of (**A**) regulatory B cells and (**B**) regulatory T cells in bronchoalveolar lavage fluid in rats after CA/CPR.

TNF-α, IFN-γ, IL-1α, IL-1β, IL-2, IL-6, and IL-10 levels increased as early as 2 or 4 h after CA with CPR and were markedly elevated at 12 h (Figure 6). In addition, the downstream genes in the MyD88-dependent pathway, IL-1β and IL-6, showed significant increases at 2, 4, and 12 h and from 2 to 12 h, respectively, after CA with CPR compared with those in the control group. These results indicated that the MyD88-dependent pathway was activated as early as 2 h after CA with CPR in this experimental model. The proportion of Bregs decreased as early as 2 h after CA with CPR. In contrast, the proportion of Tregs increased between 2 and 4 h but declined after 6 h (Figure 7). These findings suggest that Bregs and Tregs play critical regulatory roles in the post-resuscitation injury response.

## 3. Discussion

Although basic CPR is crucial for CA, it is often overshadowed by new advances in technology and pharmacology, resulting in suboptimal performance [9]. Previous studies have shown that lung injury may occur after CA and CPR in animal models [10,11]. Recent animal and clinical studies have suggested that rapid changes in intrathoracic pressure during chest compression and decompression may lead to the development of acute pulmonary edema, a phenomenon recently termed CPR-associated lung edema (CRALE) [12]. Our findings highlight the importance of pulmonary complications during CPR. Pulmonary hemorrhage was consistently observed 4 and 6 h after CA, followed by CPR in rats, accompanied by structural abnormalities in the alveolar walls, indicating the onset of acute lung injury. This histopathological damage suggests that CPR-induced mechanical stress and ischemia–reperfusion injury contribute significantly to pulmonary complications. Clinically, cardiac arrest can be caused by pulmonary or cardiogenic issues. Pediatric CA is commonly caused by asphyxia, while adult CA is often caused by cardiogenic factors. Therefore, we designed the rodent model of CA by asphyxia to further study lung injury after CA and CPR. Further studies may be needed to explore how different cardiac arrest models affect pulmonary responses, such as electrically induced ventricular fibrillation or potassium-induced arrest models.

MyD88 is a key adaptor protein involved in innate immune signaling, particularly in the pathogenesis of lung injury and pulmonary fibrosis. It modulates downstream inflammatory responses by regulating the expression of pro-inflammatory cytokines such as TNF-α, IL-1β, IL-6, and transforming growth factor-beta [13]. Our study found that a marked increase in both the mRNA and protein levels of MyD88 occurred in lung tissues following CA with CPR. In this study, cytokine levels in BALF were measured and found to be elevated, including those of TNF-α, IFN-γ, IL-1α, IL-1β, IL-2, IL-6, and IL-10. Elevated levels of circulating cytokines, which can be induced by various therapies, infections, or autoimmune conditions, may result in a cytokine storm, which is a potentially life-threatening systemic inflammatory syndrome [14]. The present findings showed that elevated cytokines may occur after CA with CPR. Interestingly, cytokine levels decreased after 12 or 24 h. Cytokines are key mediators of the immune system that play crucial roles in the regulation of inflammation. Pro-inflammatory cytokines such as IL-1α, IL-1β, TNF-α, IFN-γ, and IL-6 are particularly important, as they initiate and amplify inflammatory responses [15,16]. IL-2, which promotes T cell proliferation and immune regulation, also acts as an inflammatory cytokine in tissues such as the skin and lungs [17]. IL-10 and IL-4 are generally considered anti-inflammatory, contributing to immune regulation and limiting excessive inflammation [18]. However, IL-4 may exert detrimental effects during tissue repair by promoting pulmonary fibrosis through fibroblast activation and collagen deposition [19]. IL-8, a potent neutrophil attractant and activator, plays a significant role in ALI by mediating neutrophil recruitment and activation at the injury sites [20,21].

**Figure 8:**
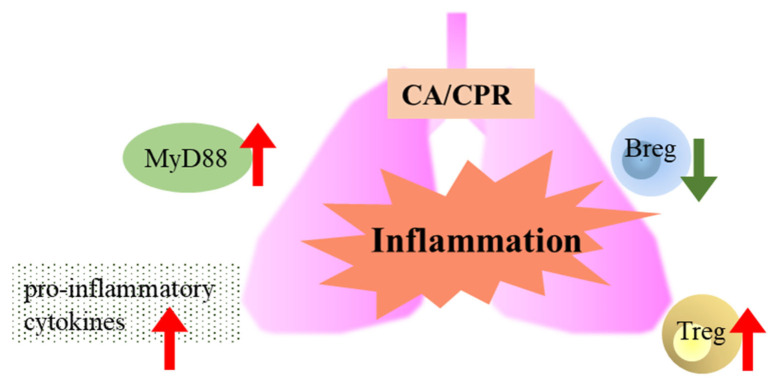
Lung inflammation, along with changes in MyD88 signaling, pro-inflammatory cytokines, and regulatory immune cells (Bregs and Tregs), occurred following CA/CPR in rats.

Bregs and Tregs are important immunoregulatory cells that help maintain immune tolerance and suppress inflammatory autoimmune responses [22,23]. In this study, the number of Tregs increased between 2 and 4 h after CA with CPR, suggesting that the inflammatory response may be suppressed. In contrast, Bregs decreased following CA with CPR, which may indicate that Tregs alone may be sufficient to maintain the immune balance. It is also possible that Bregs migrate to other organs or that B cells in the lungs differentiate into other cell types, such as memory B cells, plasmablasts, or plasma cells [24]. In summary, as shown in Figure 8, the lungs exhibit inflammation via the MyD88 signaling pathway between 6 and 12 h and produce cytokines from 2 to 12 h after CA with CPR. Meanwhile, Tregs increased in CA with CPR. We observed that MyD88 signaling and Tregs appear to contribute to immune regulation during the first 12 h after CA with CPR; however, these findings do not establish a causal role for MyD88 in pulmonary injury or immune dysregulation. Future studies using MyD88 knockout models or specific inhibitory approaches are warranted to clarify its role.

## 4. Materials and Methods

### 4.1. Animal Preparation and CA/CPR Model

Male Wistar rats (6–8 weeks old) were supplied by Biolasco Co., Ltd. (Taipei, Taiwan). All experimental protocols were reviewed and approved by the Institutional Animal Care and Use Committee of Chang Gung Memorial Hospital (Permit Number: 2022091503). This randomized study was performed on male Wistar rats weighing 250–350 g, with 6 rats assigned to each experimental group. All animals had free access to water and a standard diet. The rats were anesthetized using isoflurane inhalation. After short-term inhalation, arterial cannulation was performed on the rat’s tail for blood pressure measurement. Cardiac rhythm was monitored using leads with subcutaneous needles. Electrocardiography and arterial blood pressure data were recorded using a computerized acquisition system (MP45, Biopac, Goleta, CA, USA).

CA was induced by blocking the rat’s respiration using a sealed mask and confirmed by an abrupt decrease in blood pressure accompanied by the loss of arterial pulse. Following the onset of global ischemia or asystole, basic life support was initiated, including oxygen supply (1 L/min via an anesthesia mask), and cardiac massage was performed at 200 compressions per minute using a finger. Return of spontaneous circulation (ROSC) was defined as the presence of electrocardiographic activity with visible cardiac contractions and restoration of mean arterial blood pressure above 60 mmHg. Upon achieving ROSC, chest compressions were immediately stopped. If ROSC was not achieved within 30 min of CPR, resuscitation efforts were discontinued. The control group did not undergo any procedures to maintain normal physiological conditions. The experimental groups were sacrificed 2, 4, 6, 12, 24, and 72 h after ROSC, and samples were collected for analysis at each time point.

### 4.2. Bronchoalveolar Lavage Fluid (BALF) Harvest

BALF was harvested via endotracheal tubes, after which systemic perfusion with phosphate buffer was performed. Lungs were harvested for histological and biomolecular analyses.

### 4.3. BALF Cytokines Measurement

For the determination of cytokine levels, BALF was aliquoted and stored at −80 °C until assayed. The concentrations of IFN-γ, IL-1α, IL-2, and IL-4 were measured by cytometric bead array immunoassay using the BD™ CBA Mouse/Rat Soluble Protein Master Buffer Kit (558266, Becton Dickinson, Franklin Lakes, NJ, USA), according to the manufacturer’s instructions. Data were analyzed using flow cytometry (FACSCanto, Becton Dickinson) with FCAP Array™ software version 3.0.1 (BD Biosciences, Franklin Lakes, NJ, USA). The TNF-α, IL-1β, IL-6, IL-8, and IL-10 concentrations were measured by enzyme-linked immunosorbent assay (E-EL-R0019, E-EL-R0012, E-EL-R0015, E1167RA, and E-EL-R0016, respectively).

### 4.4. Real-Time Polymerase Chain Reaction (q-PCR) Analysis

The RNA was extracted using an RNA Isolater Total RNA Extraction Reagent (Vazyme, Nanjing, China) and then reverse transcribed using HiScript II Q RT SuperMix for qPCR (+g DNA wiper) (Vazyme, CN) at 50 °C for 15 min and 85 °C for 5 s, according to the manufacturer’s instructions. Total RNA (1000 ng) was reverse transcribed, and the resulting cDNA was amplified using specific 20× gene expression assays for Myd88 and Actb using SYBR Green (Topgen Biotechnology, Kaohsiung City, Taiwan). Primer sequences are listed in Table 1. qPCR was performed with ChamQ Universal SYBR qPCR Master Mix (Vazyme, CN) in a 10 μL reaction on the StepOne Plus Real-Time PCR System (Applied Biosystems, Foster City, CA, USA) in accordance with the manufacturer’s instructions. Specimens containing 10 ng of cDNA were amplified in triplicate with appropriate non-template controls. Amplification data were normalized to Actb expression. Quantification of relative expression [reported as arbitrary units (a.u.)] was performed using the 2^−ΔΔCt^ relative quantification method. The qPCR data were required to show a coefficient of variation of Ct values less than 2% of the mean values.

### 4.5. Histology and Immunohistochemistry (IHC) Analysis

Lung tissue morphology was first evaluated by hematoxylin and eosin (H&E) staining. MyD88 protein expression in rat lung tissue was analyzed by IHC staining using anti-MyD88 antibody (Santa Cruz Biotechnology, Dallas, TX, USA). MyD88 protein expression in lung tissues was identified by brown staining and quantified using ImageJ software version 1.53e (National Institutes of Health, Bethesda, MD, USA). All histological evaluations were performed in a blinded manner by investigators unaware of the experimental groups.

### 4.6. Western Blotting

For Western blotting, lung tissues were homogenized in RIPA buffer (ABC-AI0011, Ablebio Chemical, Seoul, Republic of Korea) using a sonicator. Protein concentrations were determined by BCA protein assay (23225, Pierce, Appleton, WI, USA). Equal amounts of protein (40 µg) were loaded onto a 12% acrylamide gel and separated by electrophoresis. Proteins were then transferred to PVDF membranes (IPVH00010, Millipore, Burlington, MA, USA) and blocked with 5% skim milk. Membranes were incubated overnight at 4 °C with primary antibodies against MyD88 (1:1000, Elabscience, Houston, TX, USA) and β-actin (1:5000, Elabscience). After washing, membranes were incubated with HRP-conjugated secondary antibody (Goat-anti-Rabbit, 1:1000, Elabscience) at 37 °C for 2 h. Protein signals were visualized using enhanced chemiluminescence (ECL, E-BC-R347, Elabscience).

### 4.7. Determination of Rat Peripheral and BALF CD4 + CD25 + Foxp3 + Tregs

BALF was centrifuged at 400 g RCF for 10 min and resuspended in Cell Staining Buffer (420201, Biolegend, San Diego, CA, USA). The prepared cells were labeled with fluorochrome-conjugated antibodies for 20 min. Tregs were labeled with anti-rat CD4 monoclonal, APC-conjugated antibody (E-AB-F1105UE-50, Elabscience), and FITC anti-rat CD25 antibody (202103, Biolegend). To detect the intracellular marker Foxp3, samples were treated with fixation buffer (420801, BioLegend) and permeabilization buffer (421002, BioLegend) and then stained with PE anti-mouse/rat/human FOXP3 Antibody (320008, BioLegend). The numbers of Tregs were determined by flow cytometry (Novocyte 3000, ACEA, Rome, Italy).

### 4.8. Determination of Rat Peripheral and BALF CD19 + CD24 + CD38 + Bregs

BALF Breg cells were labeled with CD19 polyclonal antibody, PE-conjugated antibody (BS-4755R-PE, Bioss, Woburn, MA, USA), CD38 polyclonal antibody, AF488-conjugated antibody (BS-0979R-A488, Bioss), and APC anti-rat CD24 antibody (130-106-217, Miltenyi Biotec, Bergisch Gladbach, Germany). The numbers of Bregs were determined by flow cytometry (Novocyte 3000, ACEA).

### 4.9. Statistical Analysis

The experimental rats were divided into 2 groups: CA with CPR and a sham control group. Statistical analyses were performed using the Mann–Whitney U test and the Kruskal–Wallis test. Data are presented as mean ± standard deviation (SD). *p*-values of less than 0.05 were considered significant. Statistical analyses were performed with SPSS software (version 15.0, SPSS, Inc., Chicago, IL, USA).

## 5. Conclusions

MyD88 signaling appeared to be activated as early as 2 h after CA with CPR in rats, which may be associated with early pulmonary inflammation and immune modulation. This transient increase in Tregs may reflect a short-term compensatory mechanism modulating inflammation.

## Figures and Tables

**Table 1:** Primer sequences of the Actb and Myd88 genes.

Gene Primer	Species	Sequence(5′–3′)	RefSeq	Amplicon Length	Exon Boundary
**Actb-F**	Rat	AGGCCCCTCTGAACCCTAAG	NM_031144.3	96 bp	E3-E4
**Actb-R**	Rat	CAGCCTGGATGGCTACGTACA			
**Myd88-F**	Rat	GGCAGGCTGCTAGAGTTGCT	NM_198130.1	110 bp	E1-E2
**Myd88-R**	Rat	TGTTTCTGCTGGTTGCGTATG			

## Data Availability

The original contributions presented in this study are included in the article. Further inquiries can be directed to the corresponding author.

## Abbreviations

ALI	acute lung injury
BALF	bronchoalveolar lavage fluid
Bregs	regulatory B cells
CA	cardiac arrest
CPR	cardiopulmonary resuscitation
IHC	immunohistochemistry
IL	interleukin
MyD88	myeloid differentiation primary response protein 88
ROSC	return of spontaneous circulation
TNF	tumor necrosis factor
Tregs	regulatory T cells
